# *Tc*1-like transposable elements in plant genomes

**DOI:** 10.1186/1759-8753-5-17

**Published:** 2014-06-03

**Authors:** Yuan Liu, Guojun Yang

**Affiliations:** 1 Department of Biology, University of Toronto at Mississauga, 3359 Mississauga Road, L5L 1C6 Mississauga, ON, Canada; 2 Cell and Systems Biology, University of Toronto, Toronto, Canada

**Keywords:** Transposable elements, Moss, *Tc1-mariner-IS630* superfamily, *Tc*1-like elements, *Mariner*-like elements, Plant genome, Evolution, Transposition activity

## Abstract

**Background:**

The *Tc1/mariner* superfamily of transposable elements (TEs) is widespread in animal genomes. *Mariner*-like elements, which bear a DDD triad catalytic motif, have been identified in a wide range of flowering plant species. However, as the founding member of the superfamily, *Tc*1-like elements that bear a DD34E triad catalytic motif are only known to unikonts (animals, fungi, and Entamoeba).

**Results:**

Here we report the identification of *Tc*1-like elements (TLEs) in plant genomes. These elements bear the four terminal nucleotides and the characteristic DD34E triad motif of *Tc*1 element. The two TLE families (*PpTc*1, *PpTc*2) identified in the moss (*Physcomitrella patens*) genome contain highly similar copies. Multiple copies of *PpTc*1 are actively transcribed and the transcripts encode intact full length transposase coding sequences. TLEs are also found in angiosperm genome sequence databases of rice (*Oryza sativa*), dwarf birch (*Betula nana*), cabbage (*Brassica rapa*), hemp (*Cannabis sativa*), barley (*Hordium valgare*), lettuce (*Lactuta sativa*), poplar (*Populus trichocarpa*), pear (*Pyrus x bretschneideri*), and wheat (*Triticum urartu*).

**Conclusions:**

This study extends the occurrence of TLEs to the plant phylum. The elements in the moss genome have amplified recently and may still be capable of transposition. The TLEs are also present in angiosperm genomes, but apparently much less abundant than in moss.

## Background

Transposable elements (TEs) are a major component of most eukaryotic genomes. Their transposition in genomes may lead to increase in their copy numbers. TEs are classified into two categories (Class I and Class II) based on their mechanism for transposition. Class II elements are DNA transposons that adopt a ‘cut-and-paste’ approach catalyzed by enzymes called transposases. The elements of this class are further divided into superfamilies based on different types of transposases. All of the transposases of these elements bear a DDE/D triad motif, however, different superfamilies have distinct transposases and structural features such as the length of the duplicated target site sequences [1,2]. Despite the growing number of reported active TEs, the majority of transposable elements are not active [3,4]. These elements are important for the dynamic structure of genome during evolution [5,6]. The immobilized TEs can serve as raw genetic materials for genome tinkering [7-15]. Autonomous TEs encode and produce transposases for their mobilization. Non-autonomous elements have lost their ability to encode functional transposases and rely on other sources of transposases for transposition. An ultimate group of non-autonomous elements is miniature inverted-repeat transposable elements (MITEs). They are short elements and have high copy numbers [16-18].

*Tc1-mariner-IS630* is a Class II TE superfamily first identified in nematode and insect genomes [19]. The superfamily was named after *Tc1* in *Caenorhabditis elegans*[20], and *mariner* in *Drosophila mauritiana*[21]. This superfamily is characterized by two terminal inverted repeats (TIRs) of typically 12 to 28 nt flanked by dinucleotide target site duplications (TSDs) of ‘TA’. The transposases of this superfamily contain a triad catalytic motif consisted of two aspartic acid (D) residues and a glutamate residue (E) in *Tc*1-like elements (TLEs) or aspartic acid (DDD) in *Mariner*-like elements (MLEs) and *pogo*-like elements [22,23]. The pocket formed by these residues contains the metal ions needed in the DNA cleavage reaction during transposition [24]. Based on the number of residues between the second and third catalytic residues of the DDE/D motif, *Tc1/mariner* catalytic domains can be DD34E, DD34D, DD31-33D, DD35E, DD37D, DD37E, or DD39D, each defining a subgroup of the *Tc1/mariner* superfamily [18,22,25-27]. *Tc1/mariner* elements have been considered to be confined to animals until the recent identification of DD39D *mariner*-like elements and *pogo*-like elements in plants [18,22,23]. *Tc*1-like elements are the founding subgroup of the *Tc1/mariner* superfamily and they bear the DD34E triad catalytic motif [20]. Previous studies have identified TLEs in a variety of animals and fungi [23] as well as in the parasitic amoebozoa Entamoeba invadens [28]. However, to the best of our knowledge, there has been no report of TLEs outside the unikonts (animal, fungi, and amboebozoa) [29]. Previous studies have identified TLEs in a number of animal or fungal genomes, some have been demonstrated to be active, including *Tc1* and *Tc3* in *C. elegans*[20,30,31], *Minos* in *Drosophila hydei*[32], and *Impala* in fungus *Fusarium oxysporum*[33,34]. The reconstructed fish element *Sleeping Beauty* is also a TLE [35]. *Tc*1-like elements named *Hydargo* have been identified in Entamoeba parasites [28].

Here we report the identification of TLEs in plants. The two families of full-length TLEs in the moss (*Physcomitrella patens*) genome have multiple copies that contain an intact open reading frame (ORF). These ORFs are actively transcribed and presumably also translated into functional transposases in moss. TLEs were also found in the genome sequence databases of angiosperm plants.

## Results

### Tc1-like elements in moss

*Mariner*-like elements are widespread in plant genomes [18,36]. To investigate whether plant genomes contain TLEs, moss genome sequence databases were screened because mosses are among the first terrestrial plants. When the sequence of *Tc1* transposase was used as the query sequence for BLAST search against the moss (*Physcomitrella patens*) genome database that has a coverage of approximately 8.6X [37], 118 high scoring hits (e-value: <e-8) were obtained. Close inspection of the output revealed two groups of elements that have complete terminal inverted repeats (TIRs) with terminal 5′-CAGT … ACTG-3′ sequences flanked by TSDs of dinucleotide ‘TA’. Both groups of elements contain open reading frames for transposases bearing a DD34E motif. These characteristics suggest that these two groups are TLEs and were designated as *PpTc*1 and *PpTc*2. Neither of the two families has been previously described or annotated [37]. No similar elements or their transposase sequences were found in the genome of the spike moss *Selaginella moellendorffii*.

**Figure 1:**
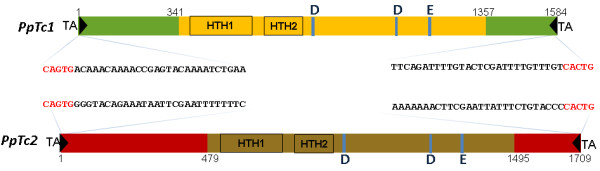
***Tc1*-like elements in the moss genome.** Schematics of *PpTc*1 and *PpTc*2 element structures. Black triangles, TIRs; regions in green or red, non-coding sequences; regions in yellow or brown, open reading frames; HTH, helix-turn-helix DNA binding motif; DDE, catalytic DD34E triad motif.

The full-length *PpTc*1 elements are 1,584 bp long with TIRs of 33 bp. It has an ORF of 338 aa with two helix-turn-helix domains and a catalytic DD34E domain (Figure 1). A total of 85 copies were retrieved from the *P. patens* genome sequence database. Among them, 75 were full length bearing the intact ends with average sequence identity of 96.3%, and 52 of which were highly similar copies with >98% sequence identity, but there were no identical copies. Nine copies were found to carry an intact full-length ORF (338 aa). To gain insights into the insertion sites of *PpTc*1 elements, it is important to inspect the sequences homologous to the flanking sequences of *PpTc*1 insertion sites. Such sequences that do not bear the TE insertions are called related empty sites (RESs). The sequence signatures of the TE insertion sites on RESs may reflect historical transposition events. Among the 75 full length copies, RESs can be found for the flanking sequences of 42 copies with 14 of them in AT rich simple repeat flanking sequences (Additional file 1: Figure S1). Most of the 28 RESs that are not AT-rich simple repeats correspond to the sequences before insertion of elements, some (for example, that of scaffold 54) may have resulted from excision events and subsequent repairing.

The full-length *PpTc2* elements are 1,709 bp long, with TIRs of 33 bp (Figure 1). A total of 22 copies of *PpTc2* were retrieved from the genome database. The 20 full-length copies have an average sequence identity of 96.6%. *PpTc2* has eight copies bearing a full-length intact 338aa ORF. Among the 20 full-length copies, RESs can be found for the flanking sequences of three copies (Additional file 1: Figure S1). While the RES of scaffold 10 clearly represents a site before insertion of an element, that of scaffold 136 may have resulted from excision events and subsequent repairing of the excision sites. Interestingly, insertion of the *PpTc*2 in scaffold 281 is accompanied by a duplication of a microsatellite unit at the insertion site. These RESs of *PpTc* insertions sites demonstrated the genomic changes caused by the activity of these elements during evolution.

### Comparison of PpTc1 and PpTc2

**Figure 2:**
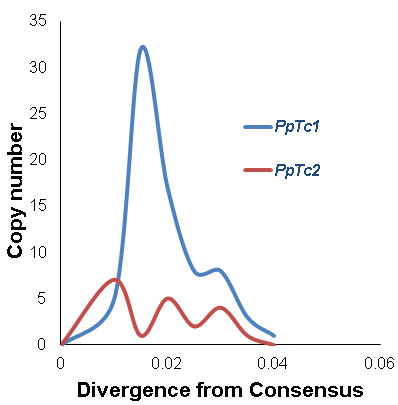
**Sequence divergence of full-length elements of *PpTc *1 and *PpTc *2.** Y-axis, number of elements; *x*-axis, level of sequence divergence from the consensus sequence of *PpTc*1 or *PpTc*2 family.

The history of activities of these elements in the genome is an important part of the evolution of these elements. According to the molecular clock theory, the mutations accumulated in each copy of an element in a TE family can be used to infer the time of divergence from their ancestral element [38]. The sequences of the ancestral element of a TE family may be approximated to the consensus sequences of the TE family. Therefore, the elements produced at the same time frame can be expected to have similar levels of sequence divergence from the ancestral element. Based on the consensus sequences of *PpTc*1 and *PpTc*2, the average sequence divergence score was calculated for each copy and the number of elements in a certain range of sequence divergence value was plotted against the sequence divergence range. The *PpTc*1 family has an average divergence value of 2.18 ± 0.08% with a significant peak at 1.5% sequence divergence (Figure 2), suggesting a recent burst of amplification events of this family occurred about 1.5 million years ago and the rate of amplification has since decreased according to a rate of 1% sequence divergence per million years. The *PpTc*2 family have an average sequence divergence value of 2.17 ± 0.20% with the most recent peak at about 1%, suggesting that *PpTc2*, similar to *PpTc*1, recently amplified about 1 million years ago. Interestingly, the *PpTc*2 dynamics is similar to the cycles of TE amplification described previously [39].

**Figure 3:**
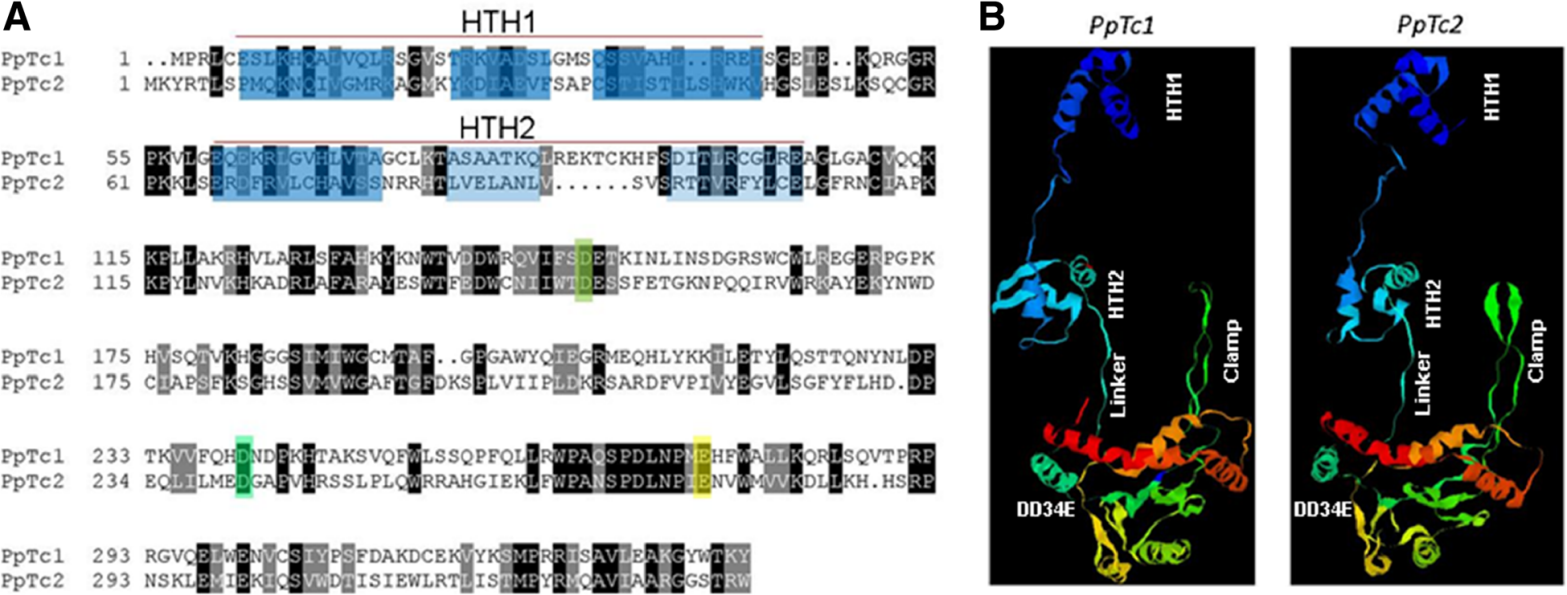
**Comparison of the putative transposases of *PpTc *1 and *PpTc *2. (A)** Alignment of peptide sequences. Colored residues: blue to cyan, α-helices of HTH motifs; green to yellow, DD34E triad motif. **(B)** Predicted three-dimensional ribbon models of transposases. Blue to red, N terminus to C terminus; HTH1 and HTH2, putative DNA binding (both) and dimerization (HTH1 only); clamp, loop structure potentially interacts with the linker of the other monomer in a transposase dimer; linker, potentially interacts with the clamp loop of the other monomer in a dimer; DD34E, catalytic active center.

Although *PpTc*1 and *PpTc*2 bear identical extreme terminal sequences ‘CAGT’ (Figure 1), their internal regions do not bear detectable DNA sequence similarities. Even the transposase coding sequences do not share significant sequence similarities between the two elements. When the putative peptide sequences of the two transposases were aligned, they share 26% (89/338) sequence identify with 47% positive (161/338) (Figure 3A). These results suggest that the two elements shared a very distant common ancestor. However, the very similar intra-family sequence divergence levels of the two families suggest that they may have invaded and amplified in the moss genome at a similar time during evolution.

Since the crystal structures of *Mos*1 and the DNA binding domain of *Tc*3 were determined, the transposase structures of *PpTc*1 and *PpTc*2 can be predicted based on these templates [24,40]. Using Phyre2 web server, the transposase structure of *Mos*1 was used by the algorithm to model the transposases. The homologous models have 100% confidence with about 95% coverage of the query sequences, suggesting highly similar protein structures between these two proteins and to the *Mos*1 transposase (Figure 3B). Based on the structural features of *Mos*1, similar features were predicted on the models of *PpTc*1 and *PpTc*2 transposases. These models provide important starting information to understand the functionality of these transposases and their structural and functional deviations from other transposases in the *Tc1/mariner* superfamily.

### Expression of PpTc1 in moss

**Figure 4:**
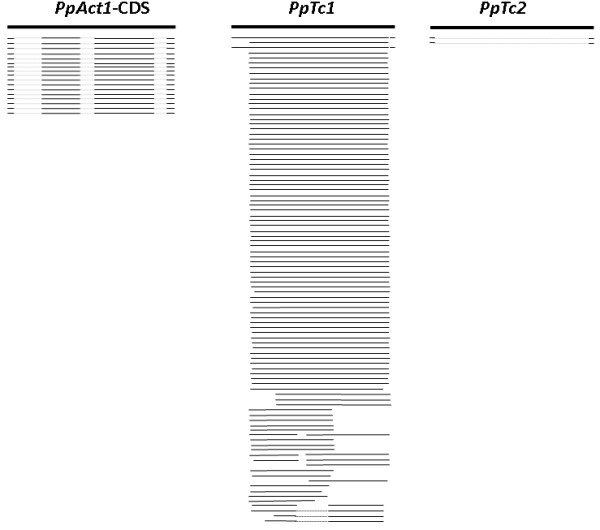
**Transcripts from *PpTc *elements.** Thick lines on top, query sequences; solid thin lines, matched regions between the queries and hits in the transcript database; dotted lines, unmatched regions reflecting intronic regions; the coding DNA sequence (CDS) of moss *actin*1 gene was used as a control.

The high intra-family sequence similarity in *PpTc*1 and *PpTc*2 and the presence of multiple copies of elements that contain intact transposase coding sequences indicate that they are potentially active. Expression of transposase is required for transposition activity, therefore it is important to determine whether *PpTc*1 and *PpTc*2 are actively transcribed. Extensive sequencing of the moss transcriptome has been previously performed and reported [41]. The expressed sequence tags derived from protonemal tissue and gametophores have been analyzed extensively and resulted in an assembled transcript database Pp0409 that contains 47,557 entries (http://www.cosmoss.org). Expressed sequence tag coverage of the genome assembly is 98% [37]. *PpTc* elements and the CDS of moss *actin*1 gene (*PpAct1*) were used to retrieve assembled transcripts from the database. Compared to the 17 transcripts from *PpAct*1, 68 assembled transcripts containing the nucleotide sequences of the ORF region were retrieved for *PpTc*1 and no transcript for *PpTc*2, suggesting that the level of transcripts of *PpTc*1 in moss cells is higher than the constitutive gene *actin*1. Each of these transcripts corresponds to a specific copy of *PpTc*1 element. Nine of the *PpTc*1 transcripts can be conceptually translated into a full-length intact transposase (Figure 4, Additional file 1: Table S1). Each of these transcripts bearing intact ORFs is derived from a specific copy of the nine genomic copies of *PpTc*1 bearing intact transposase coding sequences, suggesting that these elements are actively transcribed and yielded mature mRNA. The fact that no identical copies of *PpTc*1 were present in the genomic sequence database suggests an attenuated transposition activity after the peak amplification of the family around 1.5 million years ago. Since TE transcripts can be degraded by siRNA and their translation may be blocked by microRNAs, the *PpTc*1 transcripts were used to search against the small RNA databases [42-45]. However, no small RNA matching the coding sequences of *PpTc*1 transposase gene were retrieved, suggesting that the *PpTc*1 mRNAs are not degraded or their translation blocked, therefore may be translated into transposase proteins. Because of the abundance of the transcripts of the transposase gene, it is possible that a post-translational mechanism such as over production inhibition demonstrated for animal *Tc1/mariner* elements may have led to the repression of its transposition [46,47]. When *PpTc*2 sequences were used to search against the assembled transcript database, no transcripts were retrieved. This suggests that the expression of the transposase genes of this family is probably repressed at the transcriptional levels.

### Evolutionary relationship of transposases encoded by moss TLEs to those of animal and fungal TLEs

**Figure 5:**
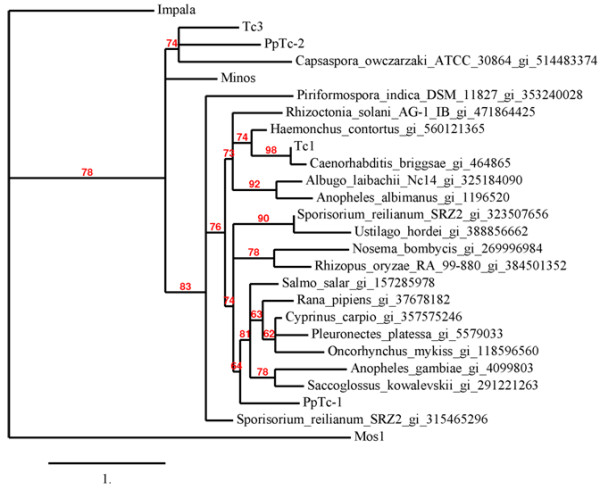
**Phylogenetic relationship of transpoases of moss TLEs to those of animal and fungal TLEs.** Names, species followed by GI numbers of each sequence; numbers on branches, percentage of bootstrap value of 1,000 reiterations.

Since TLEs have been previously described only in animal and fungal genomes, the relationship of the moss TLEs to other TLEs will help to understand the propagation of TLEs in plant genomes. Even though there are only a few well characterized TLEs in literature, recent progress in whole genome sequencing produced TLE sequences in many different genomes. Using well characterized TLE transposase sequences including *Tc*1 (X01005), *Tc*3 (P34257.1), *Minos* (CAP09075.1), and *Impala* (AF282722), together with *PpTc*1 and *PpTc*2, we retrieved representative TLE sequences in different genomes from the non-redundant protein database of Genbank. The majority of these sequences were not classified therefore named as hypothetical proteins or unknown proteins. Notably, the TLE element in *Rhizopus delemar* was found to have at least 60 copies. After removal of redundancy of sequences belonging to the same family, together with *PpTc* elements, the sequences were aligned with the previously described TLEs and a phylogenetic tree was constructed (Figure 5). Similar to that reported previously, the branches on the phylogenetic tree of these elements have relatively low bootstrap values (98% to 62%) [48]. Nevertheless, the topology of the previously analyzed elements such as *Tc*1, *Tc*3, *Impala*, and *Minos* is consistent with that shown in the previous report. *Impala* appeared to have branched off early from the rest of the TLEs. The rest of elements are grouped into two clades: *Tc1* clade and *Tc3* clade. The majority of these elements belong to the *Tc*1 clade. The fact that the phylogenetic relationship among these elements is clearly incongruent with that of their host species may suggest ancestral polymorphism or long branch attraction [49], alternatively horizontal transfer of these elements among eukaryotic species may have also contributed to the observation [50,51]. The two moss elements belong to different clades with *PpTc*1 in the *Tc*1 and *PpTc*2 in the *Tc*3 clade, further suggesting that these two elements may have different origins.

### TLEs in angiosperm genome sequence databases

**Figure 6:**
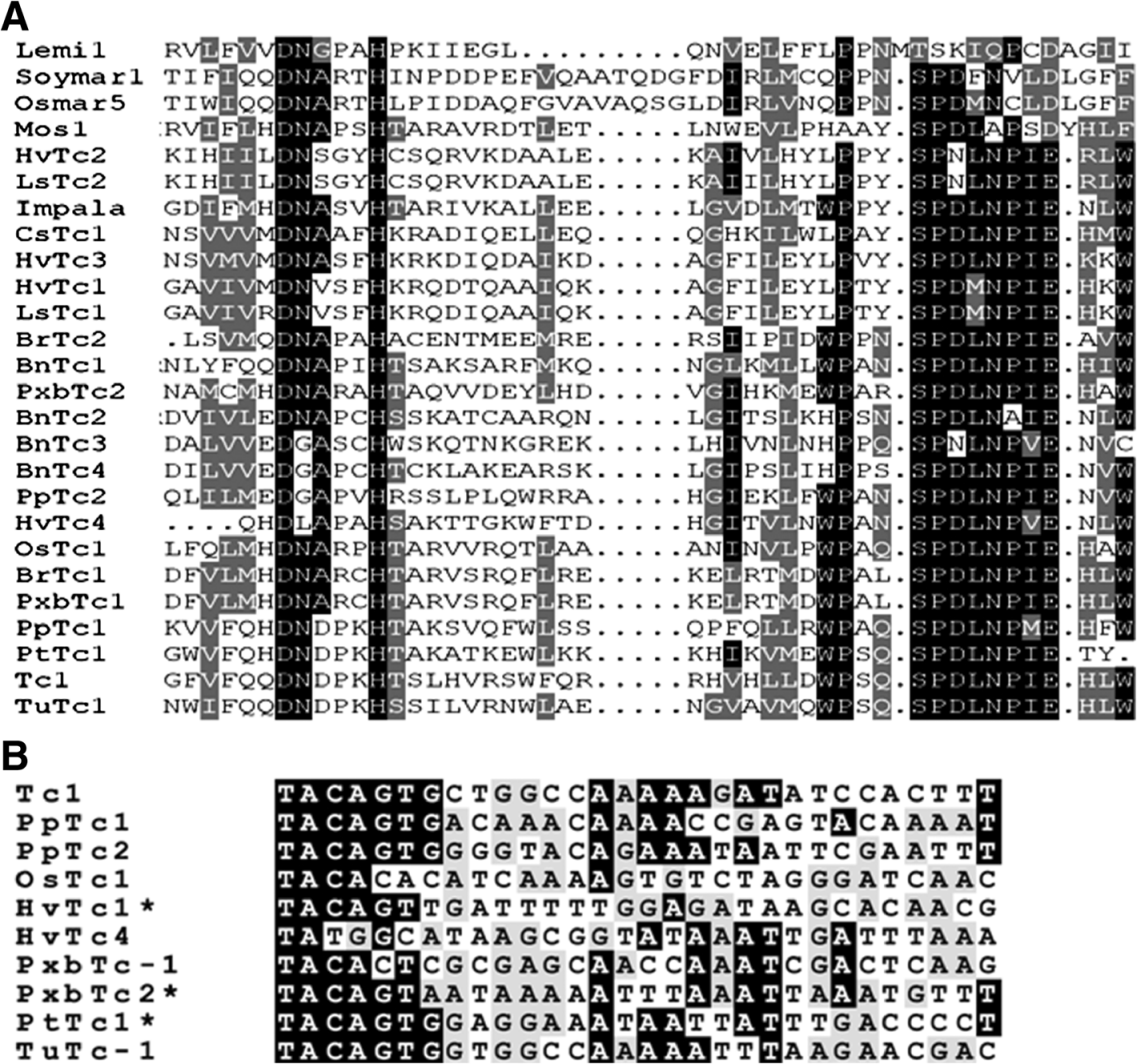
**Sequence alignment of the catalytic motifs of transposases (A) and end sequences (B) of plant TLEs. (A)** The regions containing the DDE/D catalytic motifs of the transposase sequences. Plant MLEs are shown at the bottom. **(B)** The terminal sequences of plant TLEs and *Tc*1. The degree of background shading indicates different levels of conservation of sequences. Asterisks indicate elements that only have one end present in a genomic contig. Abbreviation for species names: Os, *Oryza sativa*; Bn, *Betula nana*; Br, *Brassica rapa*); Cs, *Cannabis sativa*; Hv, *Hordium valgare*; Ls, *Lactuta sativa*; Pt, *Populus trichocarpa*; Pb, *Pyrus x bretschneideri*; Tu, *Triticum urartu*.

**Figure 7:**
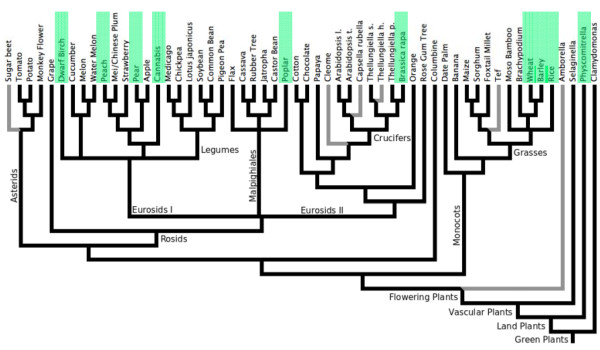
**Patchy distribution of plant species containing TLEs.** Based on the cladogram of sequenced plant genomes (up to April 2013) generated by James Schnable at CoGe (http://genomevolution.org) and used with permission. Black, published genomes; Gray, unfinished genomes; Green highlight, species containing TLEs.

To determine whether TLEs have proliferated throughout plant genomes, the predicted transposase sequences of *PpTc*1 and *PpTc2* were used as query sequences to search against all other plant genomic sequences in the GenBank WGS and NR/NT databases using TBLASTN. Segments of *Tc*1-like transposase coding sequences were identified in nine angiosperm genomes including rice (*Oryza sativa*), dwarf birch (*Betula nana*), cabbage (*Brassica rapa*), hemp (*Cannabis sativa*), barley (*Hordium valgare*), lettuce (*Lactuta sativa*), poplar (*Populus Trichocarpa*), pear (*Pyrus x bretschneideri*), and wheat (*Triticum urartu*) (Table 1). The conserved regions including at least the second (aspartic acid) and the third (glutamic acid) residues of the DD34E catalytic motif were retrieved. Most of these elements are single copies and they are not uniform in size. While TLE in the database of *Oryza sativa* is a complete element with intact terminal sequences, the majority of the plant TLEs are fragmented and do not encode a complete transposase. When the regions between the second D and the E residues of the DD34E motifs were aligned, conserved motifs surrounding these two residues were revealed (Figure 6A and Additional file 1: Figure S2). The conserved motifs surrounding the E residues of these TLEs are apparently different from those surrounding the corresponding D residue of the MLEs such as *Mos*1 (X78906), *Soymar*1 (AF078934.1), and *Osmar*5 (ACV32571.1). Among the sequenced plant genomes, the distribution of the species containing TLEs is apparently patchy (Figure 7). These results suggest that TLEs are also present in angiosperm genomes, but are much less abundant than in the moss genome.

**Table 1:** Plant Tc1-like transposases described in this study

**Element**	**Organism**	**Accession**	**ORF start**	**ORF end**	**Complete DD34E triad?**
**Plant**					Y: yes; N, no
PpTc1	*Physcomitrella patens*	ABEU01007491	7,186	8,199	Y
PpTc2	*Physcomitrella patens*	ABEU01006878	162,826	161,813	Y
OsTc1	*Oryza sativa Indica*	AAAA02041396	3,821	2,697	Y
BnTc1	*Betula nana*	CAOK01056615	1,484	1,978	N
BnTc2	*Betula nana*	CAOK01550459	168	1,214	Y
BnTc3	*Betula nana*	CAOK01014729	14,272	14,472	N
BnTc4	*Betula nana*	CAOK01486111	2	244	N
BrTc1	*Brassica rapa*	AENI01020305	162	572	N
BrTc2	*Brassica rapa*	AENI01036930	17	328	N
CsTc1	*Cannabis sativa*	AGQN01308320	302	517	N
HvTc1	*Hordium valgare*	CAJV010227559	1	1,684	Y
HvTc2	*Hordium valgare*	CAJV010272453	49	555	Y
HvTc3	*Hordium valgare*	CAJV012609061	1,716	2,114	N
HvTc4	*Hordium valgare*	CAJV011622646	1	222	N
LsTc1	*Lactuta sativa*	AFSA01593962	2	394	N
LsTc2	*Lactuta sativa*	AFSA01593962	87	485	N
PtTc1	*Populus trichocarpa*	AARH01030986	1	714	Y
PxbTc1	*Pyrus x bretschneideri*	AJSU01007483	3,055	3,606	Y
PxbTc2	*Pyrus x bretschneideri*	AJSU01007483	3,055	3,606	N
TuTc1	*Triticum urartu*	AOTI010070343	376	1,368	Y

All TLE elements bear the D34E of the DD34E catalytic motif.

## Discussion

The identification of TLEs in plant genomes expanded our knowledge on the distribution and diversity of *Tc1/mariner* elements. Elements belonging to the *mariner*-subgroup have been found to be widespread in plant genomes [18]. TLEs, however, have not been previously reported in plants. In fact, *PpTc*1 and *PpTc2* are the first *Tc1/mariner* elements described in moss. They not only expand the range of distribution of TLEs into plants, but also provide information for the development of TE-based tools for gene discovery in moss in the future.

*PpTc* elements have undergone a recent wave of proliferation. The results suggest that their transposition activities appear to have subsequently been contained in the current moss genome. Although most copies of *PpTc* elements have lost the capacity to encode a functional transposase due to mutations that interrupt the transposase coding sequences, both families have members bearing full length intact transposase-coding genes and *PpTc*1 elements are actively expressed in moss. These observations indicate that, even though the transposition activity of *PpTc1* may have been attenuated, it may still be modestly active. In addition, since the genome was sequenced with shot gun approaches, the reads for these repetitive sequences may have been misassembled. Therefore, it is possible that identical *PpTc*1 sequences are present in the genome. The absence of transcripts from *PpTc*2 may indicate a high level of repression of transposition. It remains mysterious how these elements are repressed. It is possible that, under certain environmental factors, these elements may become fully active in transposition. Alternatively, the activities of these elements may be restricted to certain tissues/organs or specific temporal stages during the life cycle of the plant. Further investigation on the repression of the transposition activities of both families will facilitate our understanding of the interaction between TEs and their host genomes.

## Conclusions

TLEs are present in plant genomes. The two families of TLEs in the moss genome have recently amplified 1 to 2 million years ago. These families contain elements that are potentially capable of transposition but their transposition activities appear to have been attenuated. TLEs were also identified in the genome databases of angiosperm plants, suggesting their distribution in multiple plant orders. The results presented in this report further our understanding of the evolutionary history of *Tc*1/*mariner* elements and provide important information for future investigations into the interaction between TEs and host genomes.

## Methods

### Retrieval of moss *Tc*1-like elements

To identify transposons related to *Tc*1-like elements, the *Tc*1 transposase peptide sequence was used as the query sequence to search against GenBank databases of *P. patens* genome with the default parameters. Each returned hit was retrieved and inspected for TIRs. Complete elements were searched against its host genome to obtain the members of its family. Nucleotide sequences of full-length TLE copies were retrieved with MITE Analysis Kit function MEMBER (http://labs.csb.utoronto.ca/yang/MAK/) [52,53]. Members of each family were retrieved with MAK with zero tolerance for end mismatches.

### Characterization of moss TLEs

Alignments of all retrieved members in each *PpTc* family were obtained with CLUSTAL, and a consensus sequence was generated. The elements were conceptually translated and scanned for long ORFs with the APE program (http://biologylabs.utah.edu/jorgensen/wayned/ape/). HTH motifs were predicted with NPS webserver (http://npsa-pbil.ibcp.fr/cgi-bin/npsa_automat.pl?page=/NPSA/npsa_hth.html) and the conserved domain database at NCBI. The putative models of *PpTc*1 and *PpTc*2 were predicted with Phyre2 (http://www.sbg.bio.ic.ac.uk/phyre2/). Sequence alignments were performed with MUSCLE at the EBI webserver (http://www.ebi.ac.uk/Tools/msa/muscle/) and the phylogenetic tree was constructed with Phylogy.fr (http://www.phylogeny.fr) with 1,000 bootstrap reiterations.

### Sequence divergence of *PpTc*1 and *PpTc*2 families

To calculate the average sequence divergence of a family, the consensus sequence of each family was constructed. The consensus sequence was used as the input for the Divergence function of MAK. Each divergence value is the complementary percentage of the similarity value in the pairwise alignment of a copy and the consensus sequence. The output contains the sequence divergence values for each member. The average divergence for each family was calculated. To plot the number of elements against divergence, values of individual divergence were grouped into bins of 0.5% and the number of elements in each bin was counted. The overall sequence similarity for a family is calculated as the complement of the average sequence divergence.

### Expression analysis of *PpTc* families

Moss TLEs *PpTc*1 and *PpTc*2 (ABEU01007491 and ABEU01006878, respectively) were used to search against the assembled transcripts database Pp0409 on the moss genome browser (http://www.cosmoss.org/) [41]. Returned hits were inspected for a long ORF that encodes a transposase bearing a DD34E catalytic motif. The loci of transcripts were cross-referenced to the nucleotide BLAST hits to remove redundancy. The sequences were also used to search for moss small RNA databases [42-45].

### Analyses of TLEs in other plant genome databases

Plant genome databases WGS and NR/NT were searched at NCBI using TBLASTN with the peptide sequences of the putative transposases of *PpTc*1 and *PpTc*2. Hits and their flanking sequences were retrieved to identify putative transposase or TIR sequences.

## Abbreviations

MLE: *Mariner*-like element; TE: Transposable element; TIR: Terminal inverted repeat; TLE: *Tc*1-like element; TSD: Target site duplication.

## Competing interests

The author(s) declare that they have no competing interests.

## Authors’ contributions

GY conceived the study. YL and GY designed and performed the analyses. YL and GY drafted the manuscript. YL and GY revised the manuscript. GY edited and finished the manuscript. Both authors read and approved the final manuscript.

## Supplementary Material

Additional file 1: Table S1 Transcripts of *PpTc1* that produce a conceptual full-length DD34E transposase. **Figure S1.** Related empty sites (RESs) for moss TLEs. **Figure S2.** Sequence alignment of plant TLEs and other *Tc1/mariner* representative elements using all predicted peptide sequences. Click here for file
