# Supplementary material for: Tc1-like transposable elements in plant genomes
Source: Mob DNA. 2014 Jun 3;5:17. doi: 10.1186/1759-8753-5-17 (PMC4054914; doi:10.1186/1759-8753-5-17)
Supplement: Additional file 1: Table S1 — Transcripts of PpTc1 that produce a conceptual full-length DD34E transposase. Figure S1. Related empty sites (RESs) for moss TLEs. Figure S2. Sequence alignment of plant TLEs and other Tc1/mariner representative elements using all predicted peptide sequences. [file 1759-8753-5-17-S1.pdf]

Supplementary Material

Table S1. transcripts of *PpTc1* that produce a conceptual full-length DD34E transposase

Accession	#EST evidence	Scaffold	Start	end	length
Pp_vt0409_37469_45516	41	89	326953	328314	1362
Pp_vt0409_1626_1984	39	107	912421	913782	1362
Pp_vt0409_33531_40740	38	67	526593	527954	1362
Pp_vt0409_23089_28041	32	34	407831	409188	1358
Pp_vt0409_3601_4394	22	121	304597	305940	1344
Pp_vt0409_26347_32004	5	4	3615886	3617201	1316
Pp_vt0409_33891_41168	41	69	294398	295759	1362
Pp_vt0409_33322_40483	2	65	1811564	1812673	1110
Pp_vt0409_15034_18253	39	226	98064	99425	1362

Fig. S1 Related empty sites (RESs) for moss TLEs

(Top sequences in each group: flanking sequences of a PpTc1 element with "||" representing a TLE element; "TA" in red, TSD; Entries below the top sequence of each group, sequences of RESs; highlighted in blue, tentative footprint; highlighted in yellow, duplication of a microsatellite unit at a *PpTc*2 insertion site).

*PpTc*1

```
scaffold_34     tattaaaaaaatacatcttattaccttatatatatatatatatatatataTA||TAtgtatatatatatatatatatatatatattacttgaaaa-aaaatccatta
scaffold_47     tattaaaaatatgtatcttatcatcttatgtatatatatctatatctatata  tatctatatctatatatatatatatatatattactttaaaataaattccatta

scaffold_34     agacttttctaatcaagaaataaaataactattagatggctgaacaTA||TAtacttggccctaaaagtaaaaaaataattataaaaaaactctagaata
scaffold_83     aggctttcctactcaagaaatagaatagcttttggatggctaaata    catacttggttctagatgtgaagaaataatt-tattggaactctagaata

scaffold_109    ttattattatatt-tttttcaggaaaaatattatatataccTA||TAtctcaaatatgttgaaatatcaagaatatccatgtatt-tttaaaatag
scaffold_61     ttatta-catattatattatagataaaatatcattaata  t   t ccttaaatatattgaaatatcaagaatattcatatattcttgataatag

scaffold_63     aaataaaataaaaataataaattaacattcttatcaaattatcaaagaTA||TAtgtatcacaaagaaccaaaaaatcaataaattttgtattactaaaaa
scaffold_78     aaataaaataagaataataaattaacactcttttcaaactataaaagaca    taaatcacaaagaaccaaaagatcaatacattcttgtttcactaaaaa
scaffold_78     aaataagacaaaaataataaattgatactcttataaaattatcaaagata    tggatcttaaaaaattgaaagatcaagatactcttgtatgacttgaaa

scaffold_288    ataaatgaaaaataggttgaataaattcagaaaaaaaacaactaggaataTA||TAcatgtgtgtgaaatgaagtatataaagaagtaagaaaattcaacttcatt
scaffold_180    attaatgaaaaataggttgaataaattcagaaaaaaa-caactaggaata    tgcatgtgtgtgaaatgaagtatatgaagaagtaagaaaagtcaacttcatt

scaffold_152    tataataaaaaaattaaaattttaattaaaacaaagttgtatgtatcTA||TAtatatttattcataaacaaaaaataagttaaaat-ttataaataaat
scaffold_2      tataa-ataaatattgaaattttaattataataaaatataatatat tca    agattt-ttaatattcaaaaaataacttcaaatattataaatatat

scaffold_69     gtgagtcaattttgtaaaaacctgggtctatttaacattcccttataTA||TAtatatatatatatataaatatatactctgtcaattgaaatagaga
scaffold_59     gtgaatcaatttaataaaaacttgggtctatttatcattcccgtata      tatatatatatatatatatata-tct---atttgatatataga

scaffold_4      ttccaagaagaataatattaaaattcatat---aat-tattttcagttgaataTA||TAtatgtatatatatatatatatatatatatatgaataa
scaffold_318    ttccaaaaattataatctcaaaatacttattccaatgtatatatatat– ata    tatatatatatatatatatatatatatgtgtataa

scaffold_4      ttatttattttctaagttttt----tttttaaagatttcatcTA||TAcatgtttttt--gtattaataaaatataattaaa---tatctaatatt
scaffold_178    ttatttattttgtaatttttttaaaattttttaaa-atttgat ta     aagttttttttagaattattaaggtttaattagattttatttaatatt

scaffold_31     ctagattcttaatctttttctaattttttataaataatatataaataTA||TAtatgcatatatttaaaaaaagcccacatctttaattctatacttttttttt
scaffold_42     ctaaatacttaataatgt-ctaatttttttataattaattt-ta            catattttttaaaaaaatccaaaatcttcaattatacacttattttt

scaffold_324    tagttatatttattaat-tttgtgttt-tatatagttatagttataTA||TAtttattaattttgtgtttt-----atatagttatatagttatatttat
scaffold_107    tatttatatttatttatatttatatttatatttatatatatttata--    tttatatatttttttattttttatttatatatatttatatttatatttat

scaffold_107    ttacaagagtatttttttatttt-atatacatacatatatatacataTA|    |TAtgtataagtgaatatagtgccaattatttttgcaaaatacacttagata
scaffold_105    ttacaagagtattttttttaatttcatatacatacatatatatatatatatatatatatgtataagtgaatatagtgccaattgtttttgcaaaatacacttagata
scaffold_107    ttacaagaat- tttttaattttatatacatacatatatatacatata        tgtgtaagtgaatacagagccaattgtttttgcaaaatacacttagatag

scaffold_67     aaaaataattaaaatattaaaataactttatttcttataaaattcctataTA||TAaac--ccatgcttactaataatatcctagcttttgttatagaagaaagaaag
scaffold_36     aaaaactattaaaatattaaaatgactttatttctttgaaaattcctatata      catccgtgtttaataataatatcctagcttttgttatagaagaaagaaag

scaffold_67     tttgataatcaaaagtaaggtttataaaatttggaatttagactataa–ttTA||TAtatataaaatttagaggatttggaattaaggtgatgaagttatagccatt
scaffold_262    tttgagaatcaaaagtaaggattataagatttagaatttaggctataatttt     tatgtaaattttagaggatttgaagttaaggtaatgaagttatagccatt
```

```
scaffold_86     gggtgggtgtttaaatgtatttattaataaacaaaatggttgggtgtgtaTA||TAtatgaataaacacacacacacacatacactcacacatatatatata
scaffold_86     gggtgggtatttaaatatatttattaataaacaaaatggttgagtgtgtata    tatgaataaacacacacacacacacacacacacacatatatatata

scaffold_96     gccatatgatttctattttt-gataataaataagttcatttaaa-gaTA||TAagtaggtattattggtcataatttttatagaattttgtcttgtatttgct
scaffold_233    gccacgtgatttttatttttggtaataaataaattcattcaaaagata    agtgtgtattatttggtggaatttttatagagttctttcttgtacttgct

scaffold_17     ttttagttattataaaatattattaatataaaaaatcaaacataTA||TAcatataagagtcatagatgaacaaccttttttcttcaagaattg
scaffold_139    ttttatgtgtcataagatattatcaatataaaaattcaaatatgta    catatagaagtcatagacaaacgacc-tttatctttaaaagttg

scaffold_54     tgaagaaattttgctaaaataaatttctaattctttcttacatatgTA||TAtgtattctttatttttttaatctatcttattttgtcttatttatggc
scaffold_382    tgaagtaattttttttaaaatcaattcctaactctctcttacatatg  cc  tatattctttatttttctaatctatcttattttgtcttatttatggc

scaffold_54     cataattcctaatcatcatagtatgataataaaaaggaataatttcataTA||TAtatattcc-tcaaatattgtgaaatatgaagaatatccaaatattctttat
scaffold_91     catgattcttattcatcacaacatgataataaaaaaaaataaatttcatatatatatatattccttcaaatatcctaaaatattaaaaatatccttatattcttgat

scaffold_41     tatatatatatatatatatatatatatatatatatatgtatgtatgtataTA||TAtgtttttatctattgaaatacatctcttattatttttttcaatcttctctt
scaffold_431    tatatatatatatatatatatatatatatatatatatatatatatatata      tatttttctgtcaaaatacatcttcaattatttttttcaacttttttctt

scaffold_122    atattgtaaaaatcctaatagtagcttcaaatccttgcttatatacaccTA||TAtgtacaattttctttatatttcacaaattaaatatttaaatatcttaaaa
scaffold_294    atattgccaaaatcctaatagtagcttcaaacccttgcttacatacaccta    tgtgtaattttcttcatatttcacaaattaaatatttaaataccataaaa

scaffold_331    ccaaaaagagtgacagtagctcccaaatcaacaccttaactttgagtactTA||TAtatctcctttatataaactccaaaccatataaaatttgtaccaaaaccat
scaffold_461    ccaaaaagagtgacaatagctcccaaatcaacaccttaactttgagcact    tgtatctccttcatataaactccaaaccatacaaaatttgtaccaaaaccat

scaffold_127    aattgaatgcagcactttcaaatcaaaggcagtaagtaagcggttttattTA||TAtagtaatggttggcaccataacttgagtattcggacaaatttttggctca
scaffold_141    aattgaaggcagcacttgcaaatcaaaggcagtaagtaagcggttttatttg    tagtaatggttggagccataacttgtgtattcggacaaatttttggctca

scaffold_127    atcatgagatatgaactattgaagtttgagtaacatttctcatgaggataTA||TAtaaggttttaagatactttatcatacatttggatattttttttggtact
scaffold_416    atcatgagatatgagctattgaagtttgaaaaacatttctcataaggatata    taaggttttaagatactttatcatacatttggatgtttttttggtact

scaffold_142    atgtacttcaattttagttgcaagatcattattttttatttagagagtttaTA||TAgttcattgaaatatatttactttgtttgta-ttttttaaattattttagat
scaffold_409    atgtaattcaattttagttgcaaagtcatcatttttacttgaatagtttgtt    gtttattaaaatatgtttgatgtatttgtagcttttttaattattttagat
scaffold_35     atataattcaattttagttacacgatcatactttttactt-gagtagtt  t gtatagtattaaaatatgtttgctttgtttgtagttttttaattattttaaat
scaffold_255    atataattcaattttagttgtaaggacatggtttttatttgaatagtttgtt    gtttattaaaatgtgtttgatgtatttataattttctaattattt

scaffold_20     aattgatcgatcaataatacgtataatgcttggtgaataaaaaccaacaTA||TAtatgccttcttgtcgatgagctccaatggtatgccattgaggttctggtt
scaffold_20     aattgatggatcaataatacgtataatgcttggtgaatcaaaaccaacata    tatgccttcttgtcgatgagctctaatggtatgccattgaggttctgatt

scaffold_8      tcatttggtaatagaatgtcctttgccctcacaaatcatggatcaatgTA||TAcattttgcaaaaaaaagtacttagcagctaattattagaatttataaagg
scaffold_4      tcatttggtaatagaatgcccttcaccctcacaaatgatggatcaatgta    cattttgcagaagaaagtacttaacagctaattgttggaatttgcaaagg

scaffold_8      tgcataaaagcgctcacacctaccggtaaggatttggatgtactagactTA||TAtgtatttctatttgacaaatagaattttagattaaactattagaaaaatt
scaffold_58     tgcataaaaactctcacacctaccaataaggatttggatgtactagactta    tgtatttttatttgactaataagattttggattaaattattaaaaaaatt
```

PpTc2

```
scaffold_136    attttat-atcacaattgaatgatttcttaagtac-atttcatgTA||TAca-tacactagcattaat----ttaatcatttaaaattacccttaa
scaffold_18     attttattaatacaattaaatgttttattaattattatttcatg    --caataaattaa-attaaccatcttaatcaattaaaattactcttaa

scaffold_10     aaggatag-ctctttatccagtactatccaatctagtaaggccaattgTA||TAgatcaggattttgaagccaatgtgatttgaattgaagaaagtgtggctgc
scaffold_90     aaggattggctctttgtccagtcctctccaaactcgtgagaccaagtg    tagagcaagctttggaagccaacttgactaggattgaagagagtgttgctgc
scaffold_211    aaggactggctctttgcccagtcctctccaagctcgtgaggccaagtg    tagagcaagctttggacgctaacttgactagaattgaagagagtgtgggtgc

scaffold_281    agcagtgttctgctttccttgtgatgatgctgatgatgctgatgatgttgTA||TAcaatatcttggtctaagctgtaacaagagcaagcactacagcacgatggt
scaffold_3      agcagtgttctgcttcccttgtgatgatgctgataatgctg---------    tacaatatcttggtctaagctgtaacaagagcaagcactacagcacgatggt
scaffold_181    agcagtgttttgcttcccttgtgatgatgctgatgatgctg---------    tacaatatcttggtctaagctgtaacaagagcaagcactacagcacaatggt
scaffold_29     agcagtgttctgttttccctgtgatgatgttgatgatgctg---------    tacaatatcttggtctaagctgtaataagagcaagcactacagcacgatggt
scaffold_283    agcaatgttctgctttccttgcgatgatgttgatgatattg---------    tacaatatcttggtctaagctgtaacaagagcaagcactacagcacgatggt
scaffold_158    agcagtgttctgcttctcttgtgatgatgctgataatactg---------    tacaatatcttggtctaagctgtaacaagagcaagcactatagcacgatggt
```

Fig. S2 Sequence alignment of plant TLEs and other *Tc1/mariner* representative elements using all predicted peptide sequences

```
Lemi1     1 ............................................................
Soymar1   1 ............................................................
Osmar5    1 MHHVAHIMHGSAHVNEEQEETELDDHEALQFELEALENDLQDYGVYAEAVNVVFDVEELP
Mos1      1 ............................................................
HvTc2     1 ............................................................
LsTc2     1 ............................................................
Impala    1 ............................................................
CsTc1     1 ............................................................
HvTc3     1 ............................................................
HvTc1     1 ............................................................
LsTc1     1 ............................................................
BrTc2     1 ............................................................
BnTc1     1 ............................................................
PxbTc2    1 ............................................................
BnTc2     1 ............................................................
```

```
BnTc3      1 ............................................................
BnTc4      1 ............................................................
PpTc2      1 ............................................................
HvTc4      1 ............................................................
OsTc1      1 ........................................MKISCQYFPYKNIQRIEDII
BrTc1      1 ............................................................
PxbTc1     1 ............................................................
PpTc1      1 ............................................................
PtTc1      1 ............................................................
Tc1        1 ............................................................
TuTc1      1 ............................................................


Lemi1      1 ............................................................
Soymar1    1 .............MQRKVKMLSNEERITIYQLLLQKSVDGKLPQGVKESVASSFSVCRKT
Osmar5    61 DSDDDQEEHANENVASKSKDLTNIQRRGIYQLLLQKSKDGKLEKHTTRLVAQEFHVSIRT
Mos1       1 ...........MSSFVPNKEQTRTVLIFCFHLKKTAAESHRMLVEAFGEQVPTVKTCER.
HvTc2      1 ............................................................
LsTc2      1 ............................................................
Impala     1 ..............MPRGKELTPSLRSRICELKKQGYSYSQIHKHFPYIPLGTIKTTCR.
CsTc1      1 ............................................................
HvTc3      1 ............................................................
HvTc1      1 ............................................................
LsTc1      1 ............................................................
BrTc2      1 ............................................................
BnTc1      1 .............MTQQYHHLTASQAHTINILTNLGHSQRTIAAQLSISQSTVSRCQRN.
PxbTc2     1 ............................................................
BnTc2      1 ................MAPLLSPRKRGKIVAHVLDGKTYKEIAQKYQVAKGTIAYTMKR.
BnTc3      1 ............................................................
BnTc4      1 ............................................................
PpTc2      1 ...............MKYRTLSPMQKNQIVGMRKAGMKYKDIAEVFSAPCSTISTILSH.
HvTc4      1 ............................................................
OsTc1     21 SATVSLLLIKTNVIMERRRHLSREEMLRAVGMIEAGSRQRTVALALNTSQSVISRLWTR.
BrTc1      1 ............................................................
PxbTc1     1 ..................MDTTPEEAAQVVALLQQGLSQRAVAAQLHLSQSAVSRVYRR.
PpTc1      1 .................MPRLCESLKHQALVQLRSGVSTRKVADSLGMSQSSVAHLRRE.
PtTc1      1 ............................................................
Tc1        1 ............................................................
TuTc1      1 ..............MGKAANLTLVEKNLIKKFRNEGKSLSEIASLVSRSKTAVFQALKP.
```

```
Lemi1      1 ................................MTGELILEK.AKETMKLVYPLQDSVKQF
Soymar1   48 IDRIWKRAKESETHDVSH....KKTKNSGRKRVEIDLSQLR.EIPLSQRTTVRTLAVAMK
Osmar5   121 VQRIWKRAKICHEQGIAVNVDSRKHGNSGRKKVEIDLSVIA.AIPLHQRRNIRSLAQALG
Mos1      49 ....WFQRFKSGDFDVDDKEHGKPPKRYEDAELQALLDEDD.AQTQKQLAE.....QLEV
HvTc2      1 ............................................................
LsTc2      1 ............................................................
Impala    46 ....REAQRGAENTTLPRSGAPRKLTEEQRDQIYDTVITDP.HVTTRDLLDSV...DNVI
CsTc1      1 ............................................................
HvTc3      1 ............................................................
HvTc1      1 ................................................KKTFYHPKADVQ
LsTc1      1 ............................................................
BrTc2      1 ............................................................
BnTc1     47 ...........PTKSRPRQSKKRTVSSQFEQAATDAYHVQS.IHNLRWASNDHIRNVLQI
PxbTc2     1 ............................................................
BnTc2     44 ......ERLHNTQKSLPTGRRPRKLSERSLRWLSREIGLFP.QSPWDYFAK.....ALSV
BnTc3      1 ............................................................
BnTc4      1 ............................................................
PpTc2     45 .....WKVHGSLESLKSQCGRPKKLSERDFRVLCHAVSSNR.RHTLVELAN.....LVSV
HvTc4      1 ............................................................
OsTc1     80 .......YRSTGTVAERHGGRYRCTTRRQDRYIQILTRRAPTITAMMLAVRLHHSSGNLI
BrTc1      1 ............................................................
PxbTc1    42 ......FQETGAFNRRPRTGRHRCTSERDDRFIVSTSLRNRHLTGVDVQQELRRVRQVAV
PpTc1     43 .........ISGEIEKQRGGRPKVLGEQEKRLGVHLVTAGCLKTASAATKQLREKTCKHF
PtTc1      1 ...........MTINRPRSGAPRKISPRGVSMILRKVKKHP.RTTWEELVNDLKLAGTTV
Tc1        1 ............................MDRNILRSAREDPHRTATDIQMIISSPNEPVP
TuTc1     46 ............VKSVLSRGRPRKTTTQCDRLMARKIKKNP.FLSSAQLQEQI...CAPV


Lemi1     28 SQGWLEKFKLRHGIKSFRRFGESGSVDTHDMEKKLEVIREKID.................
Soymar1  103 TNTSAMYRLIQSGAIKRHSSAIKPQLTEEGKRLRLEFCLSMLE..GIPHDPMFQSMYNII
Osmar5   180 VPKSTLHRWFKEGLIRRHSNSLKPYLKEANKKERLQWCVSMLDPHTLPNNPKFIEMENII
Mos1      99 SQQAVSNRLREMGKIQKVGRWVPHELNERQMERRKNTCEILLS................R
HvTc2      1 .MDSAHPTMATKVVCGWIRKGKDKPLVKTGAKTRVNVMGAIEL.................
LsTc2      1 ............................................................
Impala    98 KKRSLRYLLREMNKRKWIQK.KRVALTPLQARKRLDWAIRYQG.................
CsTc1      1 ............................................................
HvTc3      1 ..............................MPRHYGYSPKGQR................C
HvTc1     13 ARKDFQTKLQGYEACDTPIIYVDESGFAHDMPRLYGYSVKGKR................C
LsTc1      1 ..............................MPRLYGYSVKGKR................C
BrTc2      1 ............................................................
BnTc1     95 SKRRQCKLYAQEGVKKRVAV.AKPYLSHKHMQDRLLWAL.YRN.................
PxbTc2     1 .........MSHNLCCTIMMGGKEYNDNQ...............................
BnTc2     92 SESTIRREAAKMGLHKRICR.KKPFLSEKSKAARRAWAATNVD.................
BnTc3      1 ............................................................
BnTc4      1 ............................................................
PpTc2     94 SRTTVRFYLCELGFRNCIAP.KKPYLNVKHKADRLAFARAYES.................
HvTc4      1 ............................................................
OsTc1    133 SDQTVRNRLHEVNLHSRRPL.RVPPIAMHNRRIRYQWALEHRN.................
BrTc1      1 ...........................................................D
PxbTc1    96 SEWTVRRRLKEANLTPKRPA.SGPKLTAGHRQARLQFAREHLD.................
PpTc1     94 SDITLRCGLREAGLGACVQQ.KKPLLAKRHVLARLSFAHKYKN.................
PtTc1     49 TKKTIGNTLHRNGLKSCRAR.KVPLLKKAHVQARLKFANEHLN.................
Tc1       33 SKRTVRRRLQQAGLHGRKPV.KKPFISKKNRMARVAWAKAHLR.................
TuTc1     90 STRTIRRRLQELNLNSRAPR.RVPLISKQNIKNRISFAETHLS................E
```

```
Lemi1     71 ..QFPLKDVFNMDETGLFYKLQADHSLATKQLEGRKQDKERLTVVICCNADGS.....EK
Soymar1  161 HIDEKWFYMTKKSERYYLLPDEDKPHRSCKSKNFVPKVMFLTAVARPRFDSEKNVTFSGK
Osmar5   240 HIDEKWFNASKKEKTFYLYPDEEEPYFTVHNKNAIDKVMFLSAVAKPRYDDEGNCTFDGK
Mos1     143 YKRKSFLHRIVTGDEKWIFFVNPKRKKSYVDPG...Q..PATSTARPNRFGKK.....TM
HvTc2     43 ...................................ST..MKVVSARPEQVNSE.....TT
LsTc2      1 MGAIELST...............................MKVVSARPEQVNSE.....TT
Impala   140 ...IDWRRVKWSDECMVRRGQGMRPIWTFLSPR..EA..LRVQDVQEARRLGA.....VR
CsTc1      1 ..QYNWQ............................LK..NQTNAIGAIHEGKL.....FS
HvTc3     15 YGQHNWQ............................AR..GRTNIIGALLEKEL.....LT
HvTc1     57 YGQHDWH............................AK..ARTNVIGAQLNGKL.....TT
LsTc1     15 YGQHDWH............................AK..ARTNVIGAQLNGKL.....TT
BrTc2      1 ...............................................PLIDGM.....VS
BnTc1    136 WSVDNWNHVIWSDESSINLGKLAGRRYVLRNSA..NK..YNHDYIVPAYKNHK.....KS
PxbTc2    21 ...................................KNIFHKYLCFEEKVAHGG.....GS
BnTc2    134 ...QDWRRVIFTDECSVQIGEDITRHYTIRRAG..EE..YEAKHIRPIFRSGR.....TS
BnTc3      1 .....WR.....................................................
BnTc4      1 ............................................................
PpTc2    136 WTFEDWCNIIWTDESSFETGKNPQQIRVWRKAY..EK..YNWDCIAPSFKSGH.....SS
HvTc4      1 ............................................................
OsTc1    175 WAEEQWRFVCFSDESRFGMRPDTRRIRHWRTPG..RQ..QRLKSCQEVHPYSG.....GT
BrTc1      2 WSIAQWRSVLFTDECRVCLHGSDRRGRVYRRPG..ER..FAQCCFAETVAYGG.....GS
PxbTc1   138 WSIAQWRSVLFTDECRVCLHGSDRRGRVYRRPG..ER..FAQCCFAETVAYGG.....GS
PpTc1    136 WTVDDWRQVIFSDETKINLINSDGRSWCWLREG..ER..PGPKHVSQTVKHGG.....GS
PtTc1     91 DSVSDWEKVLWSDETKIELFGINPTRCVWRKKN..AA..YDPQNTVPTVKHGG.....GN
Tc1       75 WGRQEWAKHIWSDESKFNLFGSDGNSWVRRPVG..SR..YSPKYQCPTVKHGG.....GS
TuTc1    133 VSFEQWRNVLWSDESKVNLFGSDCPPRVRRPPN..TE..FDSRFTIKTVKHGG.....GS

Lemi1    124 VPLWIIGKYAKPRCFKNVNMSGLNCEY......RSNKRAWMTSVIFE.EYMRWFDNKMHG
Soymar1  221 IGIFLFVTQEPAKRTSVNRVAGTMETKAITSINRDLIRSVFIEKVLPATKEVWPRDELGS
Osmar5   300 IGIWPFTRKEPARRRSRNRERGTLVTKPI.KVDRDTIRSFMISKVLPAIRACWPREDARK
Mos1     193 LCVWWDQSGVIYYELLKPG..............ETVNTARYQQQLINLNRALQRKRPEYQ
HvTc2     61 VAFFEQLK........................................TAYPYAQ.....
LsTc2     25 VAFFEQFK........................................TAYPDAQ.....
Impala   188 QMFWAAFGHRSRTPLVPL...............VGNVNAIGIYELYS.FILPWFLQS...
CsTc1     24 VGLFDC............................KINSDVFHFWIEQ.FLIPALPE....
HvTc3     40 VCAFTS............................NINSDIFHAWITQ.DLLPKVPL....
HvTc1     82 VSTFDC............................HINSDIFHAWVMQ.DLLPKSPQ....
LsTc1     40 VCTFDC............................HINSDIFYAWVTQ.DLLPKSPP....
BrTc2      9 MTPW........................................................
BnTc1    187 VMVWGCFYGDTRGPLHYYP..............SGSIDSQKYTDMLQRSLLPIIDQDDGL
PxbTc2    41 FHMRAGISAEGQTALVPIEN.............GNLTTVRYINEVLNEHAGPFLANMGE.
BnTc2    182 LMVWGAIAYGKKWPLIRLPLSPQEVAIDGLGKGKGLNSARYIKYVLD.GPLKRCVQAHRR
BnTc3      3 ............................................................
BnTc4      1 ............................................................
PpTc2    187 VMVWGAFTGFDKSPLVIIPLDKRSA........RDFVPIVYEGVLSGFYFLHDDPE....
HvTc4      1 ............................................................
OsTc1    226 IMVWAGIWIGGRTELIWIR..............GNLNAQIYAETIVSDVIVPLQVQIGP.
BrTc1     53 CMMWAGISLEGKTALVFVPGGGRG.........GGLTADRYITDILLGHVVPYAEFVGE.
PxbTc1   189 CMMWAGISLEGKTALVFVPGGGRG.........GGLTADRYITDILLGHVVPYAEFVGE.
PpTc1    187 IMIWGCMTAFGPGAWYQI...............EGRMEQHLYKKILETYLQSTTQNYNLD
PtTc1    142 ILLLGCFSAKGTGQLIRI...............NGKMDGAMYREILNDNLLPSARKLKMG
Tc1      126 VMVWGCFTSTSMGPLRRI...............QSIMDRFQYENIFETTMRPWALQNVGR
TuTc1    184 IMVWGCFSYDGVGPLFWI...............KETMTKEIYRDILNNIMLPYAQQNMAP
```

```
Lemi1     177 R.......RVLFVVDNGPAHPKIIEGL.........QNVELFFLPPNMTSKIQPCDAGII
Soymar1   281 ........TIFIQQDNARTHINPDDPEFVQAATQDGFDIRLMCQPPN.SPDFNVLDLGFF
Osmar5    359 ........TIWIQQDNARTHLPIDDAQFGVAVAQSGLDIRLVNQPPN.SPDMNCLDLGFF
Mos1      239 KRQ....HRVIFLHDNAPSHTARAVRDTLET.....LNWEVLPHAAY.SPDLAPSDYHLF
HvTc2      76 ........KIHIILDNSGYHCSQRVKDAALE.....KAIVLHYLPPY.SPNLNPIE.RLW
LsTc2      40 ........KIHIILDNSGYHCSQRVKDAALE.....KAIILHYLPPY.SPNLNPIE.RLW
Impala    229 ........GDIFMHDNASVHTARIVKALLEE.....LGVDLMTWPPY.SPDLNPIE.NLW
CsTc1      51 ........NSVVVMDNAAFHKRADIQELLEQ.....QGHKILWLPAY.SPDLNPIE.HMW
HvTc3      67 ........NSVMVMDNASFHKRKDIQDAIKD.....AGFILEYLPVY.SPDLNPIE.KKW
HvTc1     109 ........GAVIVMDNVSFHKRQDTQAAIQK.....AGFILEYLPTY.SPDMNPIE.HKW
LsTc1      67 ........GAVIVRDNVSFHKRQDIQAAIQK.....AGFILEYLPTY.SPDMNPIE.HKW
BrTc2      13 .........LSVMQDNAPAHACENTMEEMRE.....RSIIPIDWPPN.SPDLNPIE.AVW
BnTc1     233 IENLLGDRNLYFQQDNAPIHTSAKSARFMKQ.....NGLKMLLWPAN.SPDLNPIE.HIW
PxbTc2     87 ........NAMCMHDNARAHTAQVVDEYLHD.....VGIHKMEWPAR.SPDLNPIE.HAW
BnTc2     241 ARW....RDVIVLEDNAPCHSSKATCAARQN.....LGITSLKHPSN.SPDLNAIE.NLW
BnTc3       3 ........DALVVEDGASCHWSKQTNKGREK.....LHIVNLNHPPQ.SPNLNPVE.NVC
BnTc4       1 ........DILVVEDGAPCHTCKLAKEARSK.....LGIPSLIHPPS.SPDLNPIE.NVW
PpTc2     235 ........QLILMEDGAPVHRSSLPLQWRRA.....HGIEKLFWPAN.SPDLNPIE.NVW
HvTc4       1 ............QHDLAPAHSAKTTGKWFTD.....HGITVLNWPAN.SPDLNPVE.NLW
OsTc1     271 ........LFQLMHDNARPHTARVVRQTLAA.....ANINVLPWPAQ.SPDLNPIE.HAW
BrTc1     103 ........DFVLMHDNARCHTARVSRQFLRE.....KELRTMDWPAL.SPDLNPIE.HLW
PxbTc1    239 ........DFVLMHDNARCHTARVSRQFLRE.....KELRTMDWPAL.SPDLNPIE.HLW
PpTc1     232 PT......KVVFQHDNDPKHTAKSVQFWLSS.....QPFQLLRWPAQ.SPDLNPME.HFW
PtTc1     187 R.......GWVFQHDNDPKHTAKATKEWLKK.....KHIKVMEWPSQ.SPDLNPIE.TY.
Tc1       171 ........GFVFQQDNDPKHTSLHVRSWFQR.....RHVHLLDWPSQ.SPDLNPIE.HLW
TuTc1     229 ........NWIFQQDNDPKHSSILVRNWLAE.....NGVAVMQWPSQ.SPDLNPIE.HLW

Lemi1     221 RAFKMHYRRRFYREILEGYELGQSDPGTINVLDAISFAVSAWTINVRRETILNCFRHCKI
Soymar1   332 SAIQ...SLH.......YKE.......APKTIDELVNAVVKSFENYCVVKSNFIFLSLQL
Osmar5    410 ASLQ...SLT.......HNR.......ISRNMDELIENVHKEYRDYNPNTLNRVFLTLQS
Mos1      289 ASMG...HAL.......AEQ.......RFDSYESVKKWLDEWFAAKDDEFYWRGIHKLPE
HvTc2     121 KVMN...ERV.......RNNR......FFSSAKEFRAAIAEFFDSTLAKIAPFLRGRIND
LsTc2      85 KLMN...ERV.......RNNR......FFSSAKEFRGAIAEFFDSTLAKIAPFLTGRIND
Impala    274 ALMKAEIYRL.......HPELTHTED.TVATQHALVLAAMEAWDNIEDRVLKNLCETMPN
CsTc1      96 AWVK...RKR.......KEW.......LIDSVDELFRVFFESCMNNKVF...........
HvTc3     112 AHAK...ARR.......RKE.......RCD.VDHLFSFFN....................
HvTc1     154 AQAK...ALR.......RKR.......QCD.IDTLFSAPLF...................
LsTc1     112 AQAK...ALR.......RK........RLCDIDTLFYD......................
BrTc2      57 DWMK...DYI.......QRNYPNLDGGRQRTHNSLRVIVTEAWHSVPTEFFKRLLKGMPA
BnTc1     286 HLLK...RKTWRKWHGQKRNLH.....AQVTSDDLENVVQDAWDSIDPSILVSLVLSMPS
PxbTc2    132 DERC...RPV.......RDRVL.....PPITLRGLKDALIEEWVNIPQHMLKNLV.....
BnTc2     290 DQVK...LKL.......GRMNR.....QATSLNELWEQIQQAWDEVDIGSVNRVVDSMEE
BnTc3      48 LQLK...LQV.......SCLPR.....KATTLEEL.........................
BnTc4      46 QLLK...TKV.......SQLPT.....RATNLDMLWEQVQACWADIDQGYI.........
PpTc2     280 MVVK...DLL.......KHHS......RPNSKLEMIEKIQSVWDTISIEWLRTLISTMPY
HvTc4      42 DIVK...RKL.......RDA.......RPNTLDELKAAIEASWASRTPQQ..........
OsTc1     316 DMLQ...RRA.......LPNME.....GIQSQEDLFLLLQRTWAAIPQRDLDELILSMPN
BrTc1     148 DELK...RRV.......RARNP.....VPASVDELKTALLEEWDGIPQETVKKLIRSM..
PxbTc1    284 DELK...RRV.......RARNP.....VPASVDELKTALLEEWDGI..............
PpTc1     279 ALLK...QRL.......SQVTP.....RPRGVQELWENVCSIYPSFDAKDCEKVYKSMPR
PtTc1     232 ............................................................
Tc1       216 EELE...RRL.......GGI.......RASNADAKFNQLENAWKAIPMSVIHKLIDSMPR
TuTc1     274 AEVK...KRL.......QRE.......NIKNKDQLWQEIQRTWINIPKSFCERLVMSMTS
```

```
Lemi1     281 HSD................................................
Soymar1   375 CMIETMKAKGSNRYTSQHMQKEKLETEEQLPIQLKCDPILVQETLDYLNNN
Osmar5    453 CYIEVMRANGGNRYKIPHMNKERLEALGVLPKALSCDRGLYERVMESLAN.
Mos1      332 RWEKCVASDGKYFE.....................................
HvTc2     165 NFQTI..............................................
LsTc2     129 NFQTI..............................................
Impala    326 RVTAVITAEGWYTKY....................................
CsTc1         ...................................................
HvTc3         ...................................................
HvTc1         ...................................................
LsTc1         ...................................................
BrTc2     107 RCQ................................................
BnTc1     338 RLEAVIKAKGGHTKY....................................
PxbTc2    172 ..EAVLRARGGNTKY....................................
BnTc2     335 RRRDVVAAKGSYTRF....................................
BnTc3         ...................................................
BnTc4         ...................................................
PpTc2     324 RMQAVIAARGGSTRW....................................
HvTc4         ...................................................
OsTc1     361 RCSCVVSVRGRNTDY....................................
BrTc1         ...................................................
PxbTc1        ...................................................
PpTc1     324 RISAVLEAKGYWTKY....................................
PtTc1     232 ........GGSSSSE....................................
Tc1       259 RCQAVIDANGYATKY....................................
TuTc1     317 RCVEVLRNKGGATKY....................................
```
